# Combined immune checkpoint protein blockade and low dose whole body irradiation as immunotherapy for myeloma

**DOI:** 10.1186/s40425-014-0043-z

**Published:** 2015-01-20

**Authors:** Weiqing Jing, Jill A Gershan, James Weber, Dominique Tlomak, Laura McOlash, Catherine Sabatos-Peyton, Bryon D Johnson

**Affiliations:** Division of Hematology/Oncology/Transplant, Department of Pediatrics, Medical College of Wisconsin, Milwaukee, WI 53226 USA; Novartis Institutes for BioMedical Research, Inc., Cambridge, MA 02139 USA

**Keywords:** Low dose whole body irradiation, Immune checkpoint proteins, Blockade, PD-L1, TIM-3, LAG-3, CTLA4, 2B4, Myeloma

## Abstract

**Background:**

Multiple myeloma is characterized by the presence of transformed neoplastic plasma cells in the bone marrow and is generally considered to be an incurable disease. Successful treatments will likely require multi-faceted approaches incorporating conventional drug therapies, immunotherapy and other novel treatments. Our lab previously showed that a combination of transient lymphodepletion (sublethal whole body irradiation) and PD-1/PD-L1 blockade generated anti-myeloma T cell reactivity capable of eliminating established disease. We hypothesized that blocking a combination of checkpoint receptors in the context of low-dose, lymphodepleting whole body radiation would boost anti-tumor immunity.

**Methods:**

To test our central hypothesis, we utilized a 5T33 murine multiple myeloma model. Myeloma-bearing mice were treated with a low dose of whole body irradiation and combinations of blocking antibodies to PD-L1, LAG-3, TIM-3, CD48 (the ligand for 2B4) and CTLA4.

**Results:**

Temporal phenotypic analysis of bone marrow from myeloma-bearing mice demonstrated that elevated percentages of PD-1, 2B4, LAG-3 and TIM-3 proteins were expressed on T cells. When PD-L1 blockade was combined with blocking antibodies to LAG-3, TIM-3 or CTLA4, synergistic or additive increases in survival were observed (survival rates improved from ~30% to >80%). The increased survival rates correlated with increased frequencies of tumor-reactive CD8 and CD4 T cells. When stimulated in vitro with myeloma cells, CD8 T cells from treated mice produced elevated levels proinflammatory cytokines. Cytokines were spontaneously released from CD4 T cells isolated from mice treated with PD-L1 plus CTLA4 blocking antibodies.

**Conclusions:**

These data indicate that blocking PD-1/PD-L1 interactions in conjunction with other immune checkpoint proteins provides synergistic anti-tumor efficacy following lymphodepletive doses of whole body irradiation. This strategy is a promising combination strategy for myeloma and other hematologic malignancies.

**Electronic supplementary material:**

The online version of this article (doi:10.1186/s40425-014-0043-z) contains supplementary material, which is available to authorized users.

## Background

Reports of immunotherapy-induced clinical responses have brought the study of tumor immunity front and center in the mission to eliminate cancer. Central to tumor immunity is the killing potential of activated tumor-specific T cells. Tumor-specific T cells can be detected in both preclinical animal models and cancer patients, but due to multiple immune suppressive factors within the tumor microenvironment, T cells fail to maintain an activated state against progressing tumor and are rendered tolerant or exhausted. T cell activation is initiated through tumor antigen recognition by the T cell receptor (TCR) and is regulated by a balance of activation and inhibitory intracellular signals. These signals are initiated by engagement of co-stimulatory and co-inhibitory receptors with their cognate ligands. One of the promising approaches to induce and maintain tumor-specific T cells in an activated state is to interfere with signaling through inhibitory (also referred to as immune checkpoint) receptors.

There are multiple known T cell checkpoint receptors, and there is evidence that blocking interaction of these receptors with their respective ligands can increase anti-tumor immune responses. One of the most studied checkpoint receptors is CTLA4. Cell surface CTLA4 expression is rapidly upregulated when T cells are activated, and it is constitutively expressed on Foxp3^+^ regulatory T cells [1]. Signaling through CTLA4 arrests T cell activation by outcompeting co-stimulatory receptors (CD80 and CD86) for binding to CD28. CTLA4 binding to CD28 results in reduced T cell survival, cytokine production and T cell cycle arrest [2]. Testament to the importance of CTLA4 in dampening T cell activation is the occurrence of a lethal polyclonal lymphoproliferative disease that occurs in CTLA4 knockout mice [3]. Antagonistic anti-CTLA4 antibodies have been extensively tested in cancer models as a strategy to activate anti-tumor immunity, and CTLA4 was the first immune checkpoint targeted in the clinic for cancer therapy. The anti-tumor effects associated with blocking CTLA4 in vivo have been shown to involve depletion of regulatory T cells as well as restoring effector T cell function [4,5]. Notably, CTLA4 blockade results in increased ratios of effector CD8 T cells to regulatory T cells in tumors, possibly due to higher levels of CTLA4 expression by regulatory T cells [4]. In 2010, a phase III randomized controlled clinical trial showed prolonged survival of metastatic melanoma patients when treated with the anti-CTLA4 antibody ipilimumab [6]. In melanoma patients, blocking CTLA4 produced a host of immune-related toxic side effects (referred to as immune-related adverse events). However, based on the promising responses in melanoma patients, ipilimumab was the first checkpoint-blocking antibody to be FDA approved (for the treatment of melanoma).

Our laboratory has focused on blocking the checkpoint receptor programmed death receptor-1 (PD-1) pathway in the treatment of myeloma. PD-1 (CD279) is an immunoglobulin superfamily transmembrane receptor that is expressed on activated T cells, regulatory T cells, B cells and NK cells. Ligands for PD-1 include PD-L1 (B7-H1, CD274) and PD-L2 (B7-DC, CD273) [7]. PD-L2 expression is restricted to hematopoietic cells, notably myeloid cells including dendritic cells and macrophages, but PD-L1 is broadly expressed on hematopoietic and non-hematopoietic cells as well as on a variety of murine and human malignancies [8,9]. Most of data showing the anti-tumor efficacy induced by blocking the PD-1/PD-L1 inhibitory receptor axis has been generated from preclinical and clinical studies involving solid tumors. Preclinical studies have shown that blocking the PD-L1/PD-1 axis with anti-PD-L1 or anti-PD-1 antibodies promotes anti-tumor T cell responses in pancreatic carcinoma [10], B16 melanoma [11], and CT26 colon carcinoma [12]. In a recent clinical study, patients with colorectal cancer, renal cell carcinoma or melanoma showed objective responses to anti-PD-L1 therapy [13]. Patients with PD-L1^+^ tumors, but not PD-L1^−^ tumors, showed objective responses when treated with an anti-PD-1 blocking antibody [14]. Clinically, combining anti-CTLA4 (ipilimumab) with anti-PD-1 (nivolumab) antibodies resulted in even greater anti-tumor efficacy, as tumor regression occurred in 80% of patients with advanced melanoma [15]. Importantly, immune adverse events were qualitatively similar to that experienced with prior treatment of either antibody alone.

Less well-characterized T cell immune checkpoint receptors include lymphocyte activating gene 3 (LAG-3 or CD223), T cell immunoglobulin and mucin domain 3 (TIM-3), 2B4 (CD244), and others. LAG-3 is a member of the immunoglobulin (Ig) superfamily that binds to MHC class II molecules, and has recently been reported to also bind L-selectin [16]. LAG-3 is expressed on activated and tolerized T cells, NK cells, plasmacytoid dendritic cells, and regulatory T cells and it is known to negatively regulate the expansion of activated T cells [17,18]. Preclinical studies have shown that combined treatment of LAG-3 and PD-1 blocking antibodies provided a synergistic anti-tumor effect [19]. The TIM family of transmembrane receptor proteins includes several members (TIM-1, 2, 3 and 4 in mice, but only TIM-1, 3 and 4 are known to be expressed in humans). Ligation of TIM-1 regulates Th2 CD4 T cell responses, and in mice, TIM-1 promotes CD4 T cell activation [20]. TIM-3 is a checkpoint receptor that is co-expressed on PD-1^+^ CD8 T cells in mice harboring solid or hematologic malignancies [21,22]. The ligand for TIM-3 is galectin-9 which is expressed by multiple tumors. Reduced galactin-9 expression correlates with reduced disease progression in a majority of solid tumors [23]. PD-1^+^TIM-3^+^ T cells derived from patients with melanoma, non-small cell lung cancer or non-Hodgkin’s lymphoma are defective in proliferation and cytokine production [24-26]. TIM-3 is also expressed on regulatory T cells, monocytes, NK cells, and dendritic cells [27]. Data suggests that TIM-3 can play anti-inflammatory or pro-inflammatory roles in cells depending on the physiologic setting [28,29]. In a preclinical mouse B16F10 melanoma model, combined blockade of TIM-3 and PD-1, or TIM-3 and CTLA4, was more effective in prolonging survival than blocking either protein alone [30]. In addition, the combination of anti-CTLA4, anti-TIM-3 and anti-LAG-3 produced further suppression of B16F10 tumor growth [30]. These data demonstrate a mechanistic synergy when multiple inhibitory receptors are blocked.

2B4 (CD244) engagement with CD48 was originally described as facilitating CD8 T cell proliferation [31]. Recent data examining hepatitis C-virus (HCV)-specific CD8 T cells showed that crosslinking 2B4 on CD8 T cells with low versus high 2B4 expression increased or decreased proliferation, respectively, and 2B4 blockade preferentially increased proliferation of HCV CD8 T cells with high 2B4 expression [32]. Similar to other checkpoint proteins, 2B4 is upregulated on exhausted virus-specific CD8 T cells [33]. Together these data suggest that 2B4 plays a role in the regulation of CD8 T cells.

Despite the promising results afforded by blocking CTLA4 and the PD-1/PD-L1 axis in the treatment of solid tumors, targeting these checkpoints in hematologic malignancies has been relatively understudied. Multiple myeloma is a hematologic malignancy involving plasma cells. In humans, PD-L1 is expressed on CD138^+^ malignant plasma cells [34-38]. We have shown that PD-1 is upregulated on peripheral blood and bone marrow in myeloma patients up to 30 days following autologous transplant [35]. In humans, blocking the PD-L1/PD-1 axis may act to prevent inhibitory signaling when effector T cells engage with tumor cells and when T cells are undergoing homeostatic expansion.

Our preclinical studies have demonstrated improved anti-myeloma T cell immunity when the PD-1/PD-L1 axis is blocked. Using a murine model of myeloma, we showed that administration of an anti-PD-L1 blocking antibody elicits rejection of PD-L1 expressing tumor cells. When anti-PD-L1 was administered immediately following hematopoietic stem cell transplantation in combination with a tumor cell-based vaccine, myeloma was eliminated in approximately 40% of treated mice [35]. We also reported that 5T33 tumor was eliminated in approximately 50% of mice when treated with anti-PD-L1 following radiation-induced lymphopenia [39]. Based on these data and the data of others, we hypothesized that blocking a combination of checkpoint receptors in the context of lymphodepleting radiation would boost anti-tumor immunity. The results presented here confirm the hypothesis and show that PD-1/PD-L1 blockade in combination with either TIM-3, LAG-3 or CTLA4 blockade synergistically improves the survival of myeloma bearing mice.

## Results

### Increased PD-1, TIM-3, LAG-3 and 2B4 expression on CD4 and CD8 T cells directly correlates with myeloma burden

**Figure 1:**
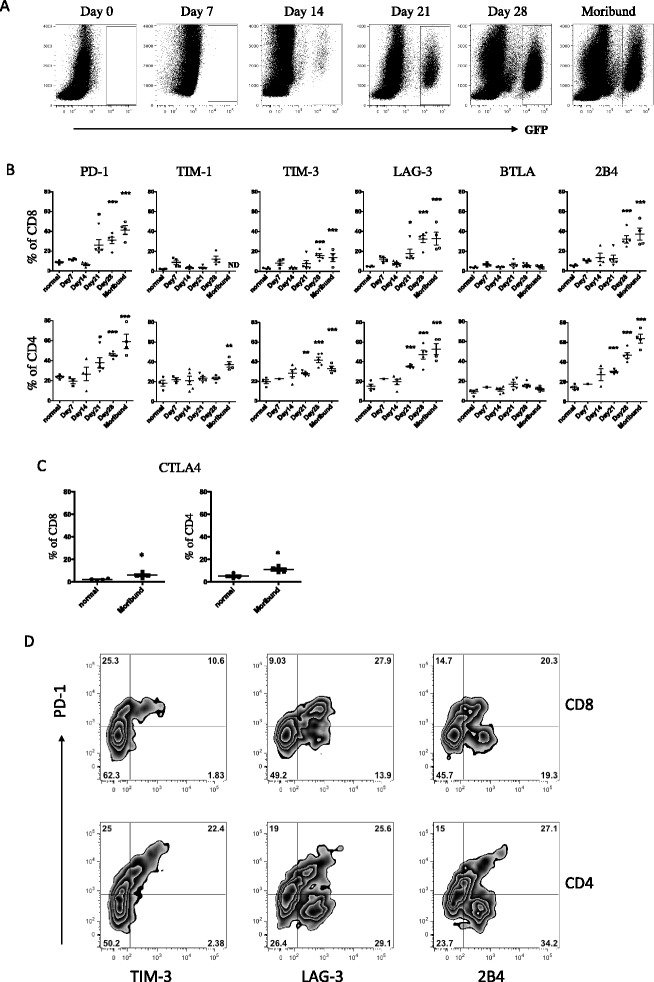
**Expression of immune checkpoint proteins on T cells in bone marrow of myeloma bearing mice over time.** KaLwRij mice were inoculated with 2×10^6^ 5T33-GFP cells iv. Myeloma bearing mice were euthanized between days 7 and 28 and when moribund at days 29-40 after myeloma injection. Femoral bone marrow cells were harvested and **(A)** GFP^+^ tumor cell accumulation was monitored by flow cytometry. CD4^+^ and CD8^+^ T cells in the bone marrow were analyzed by flow cytometry for expression of **(B)** PD-1, TIM-3, LAG-3, BTLA and 2B4 at each of the time points indicated, or for surface expression of **(C)** CTLA4 when animals were moribund. T cells harvested from naïve non-myeloma bearing mice were analyzed as controls. Immune checkpoint protein percentages were calculated based on isotype controls. **(D)** Expression of TIM-3 and PD-1, LAG-3 and PD-1 or 2B4 and PD-1 on gated CD8^+^ or CD4^+^ T cells harvested from the BM of moribund mice. Data shown are representative of more than four independent analyses. *p < 0.05, **p < 0.01, ***p < 0.001 as compared to T cells from naïve non-myeloma bearing mice.

Our previous work demonstrated that the PD-1/PD-L1 pathway is important in suppressing immune responses to 5T33 myeloma, and that PD-1 expression on T cells is related to 5T33 burden in the myeloma-resident tissues (bone marrow, spleen and liver) [35,39]. Besides expression of the immune checkpoint protein PD-1, T cells within tumor environments may develop a dysfunctional phenotype accompanied by the increased expression of other checkpoint proteins. We therefore examined CD4 and CD8 T cells for the expression of other checkpoint receptors over time in myeloma-bearing animals. GFP^+^ 5T33 cells could be observed in the bone marrow as early as 7 days after iv inoculation, with increasing accumulation of myeloma cells over time (Figure 1A). When myeloma-bearing mice became moribund (29-38 days after 5T33 inoculation), 20-35% of the cells in the bone marrow consisted of GFP^+^ tumor cells. As previously described [39], there was a significant increase in the percentages of PD-1^+^ bone marrow-derived CD4 and CD8 T cells as early as 21 days after myeloma inoculation (Figure 1B). As myeloma burden progressed, the percentages of bone marrow-derived PD-1^+^ CD4 and CD8 T cells increased to 40-70% and 30-50%, respectively. Similar to the increase in PD-1 expression, increasing percentages of T cells in the bone marrow expressed TIM-3, LAG-3 and 2B4 as myeloma burden progressed (Figure 1B). In contrast, percentages of T cells expressing TIM-1 or BTLA did not increase over time. Similar results were also observed in the spleen (Additional file 1: Figure S1). Together, these data show that there is upregulated expression of multiple checkpoint receptors on T cells in tissues where myeloma cells are present, and that an accumulation of checkpoint-expressing T cells occurs over time. In order to show that checkpoint receptors are co-expressed on T cells, CD4 and CD8 cells in the bone marrow of moribund mice were analyzed for PD-1, TIM-3, LAG-3 and 2B4 expression using multicolor flow cytometry. For CD8 T cells (Figure 1C, top row), PD-1 was co-expressed on 85% of TIM-3^+^ cells, 67% of LAG-3^+^ cells, and 51% of 2B4^+^ cells. For CD4 T cells (Figure 1C, bottom row), co-expression of PD-1 was observed on 90% of TIM-3^+^ cells, 47% of LAG-3^+^ cells, and 44% of 2B4^+^ cells. Notably, there were also PD-1^+^ cells that did not co-express the other checkpoints (upper left quadrants in Figure 1C), as well as major subsets of LAG-3^+^ and 2B4^+^ cells that did not co-express PD-1 (lower right quadrants in Figure 1C).

Next, we determined whether checkpoint ligands for TIM-3, LAG-3 and CTLA4 were expressed on the tumor and cells within the tumor microenvironment (i.e., spleen). PD-L1 is highly expressed on 5T33 myeloma cells [35], but the ligands for TIM-3 (galectin-9), LAG-3 (MHC class II), and CTLA4 (CD80) are not (Additional file 2: Figure S2A). In vitro irradiation with 500 cGy did not change the galectin-9, MHC class II or CD80 expression on 5T33 myeloma cells. Not surprisingly, galectin-9, MHC class II and CD80 is present on a variety of cells in the tumor microenvironment including B cells, macrophages, DCs and monocytes (Additional file 2: Figure S2B).

### Immune checkpoint protein expression is increased on CD8 T cells in mice treated with lymphodepleting radiation and anti-PD-L1

**Figure 2:**
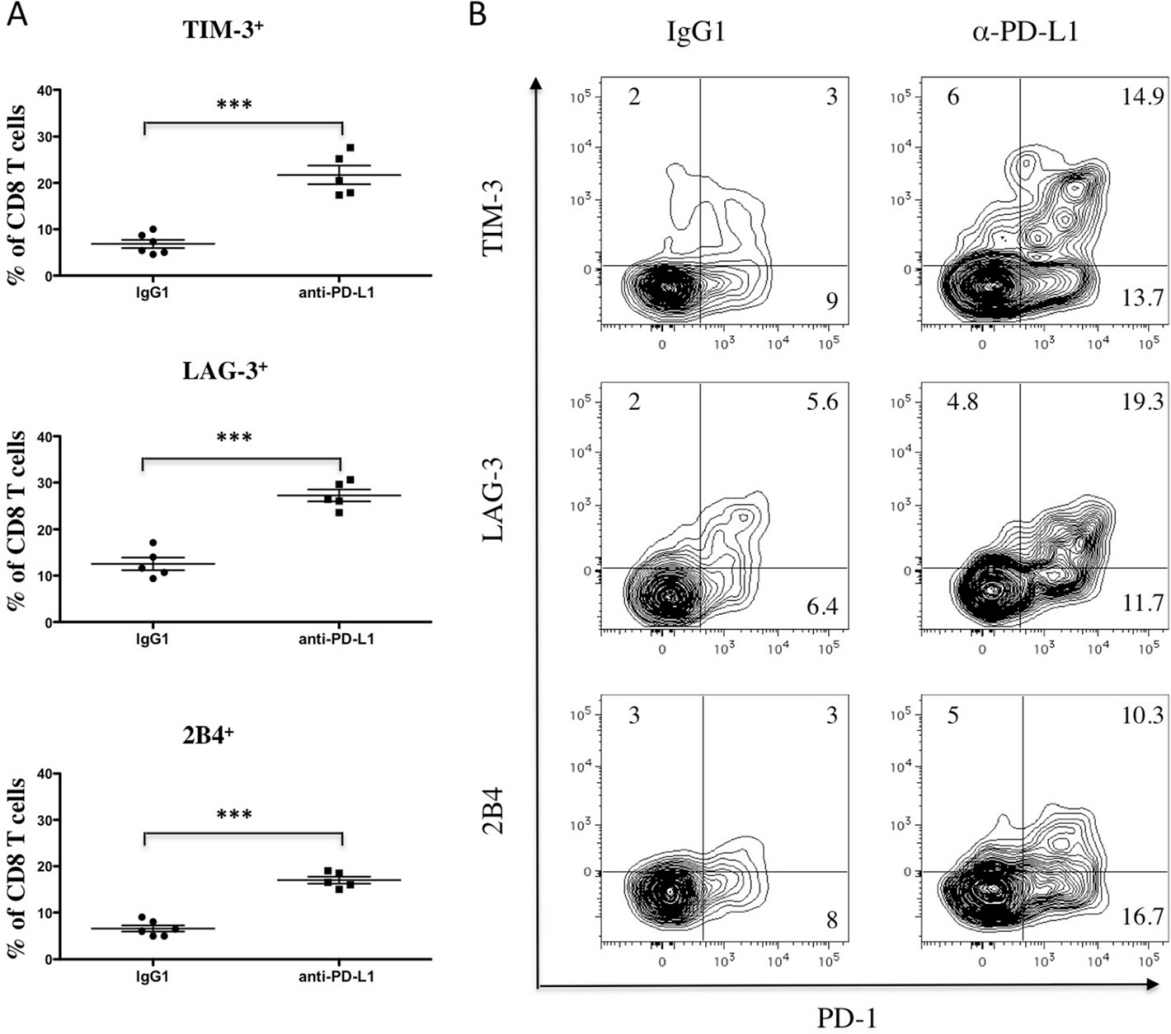
**Expression of immune checkpoint proteins are increased on T cells in mice treated with sublethal whole body irradiation and anti-PD-L1.** Myeloma bearing KaLwRij mice were treated with 500 cGy whole body irradiation 7 days after tumor cell injection. Treatment with anti-PD-L1 or control IgG (200 μg ip) was initiated 5 days later and specifically given 12, 14, and 19 days after tumor injection. Mice were euthanized at day 21, splenocytes were harvested, and the CD8 T cells were analyzed by flow cytometry for immune checkpoint protein expression. **(A)** Frequency of CD8^+^TIM-3^+^, CD8^+^LAG-3^+^ and CD8^+^2B4^+^ cells in spleens of anti-PD-L1 treated mice as compared with spleens harvested from control antibody (IgG1) treated mice. ***p < 0.001. **(B)** Expression of TIM-3 and PD-1, LAG-3 and PD-1 or 2B4 and PD-1 on gated CD8^+^ T cells. Data shown are representative of more than four independent analyses.

We previously showed that blocking the PD-1/PD-L1 axis with a PD-L1-specific monoclonal antibody synergized with lymphodepleting whole body irradiation (WBI) to facilitate a T cell-mediated anti-myeloma response [39]. To determine the influence of this treatment on T cell immune checkpoint expression, mice with established myeloma were treated with 500 cGy WBI 7 days after 5T33 inoculation, followed by three treatments with anti–PD-L1 or control IgG on days 5, 7 and 12 after WBI. On day 14 after WBI, CD8 T cells were harvested from bone marrow and analyzed for expression of PD-1, TIM-3, LAG-3 and 2B4 by flow cytometry. There were significant increases in the percentages of CD8 T cells that expressed TIM-3, LAG-3 or 2B4 in mice treated with anti-PD-L1 as compared to controls treated with IgG1 (Figure 2A). Interestingly, the mice treated with anti-PD-L1 also had a marked increase in percentages of PD-1^+^ CD8 T cells (Figure 2B), and relatively large percentages of the PD-1^+^ cells co-expressed TIM-3 (52%), LAG-3 (60%) or 2B4 (40%) (Figure 2B).

### Blocking PD-L1 in combination with TIM-3, LAG-3 or CTLA4 blockade synergize to improve the survival of lymphodepleted myeloma-bearing mice

**Figure 3:**
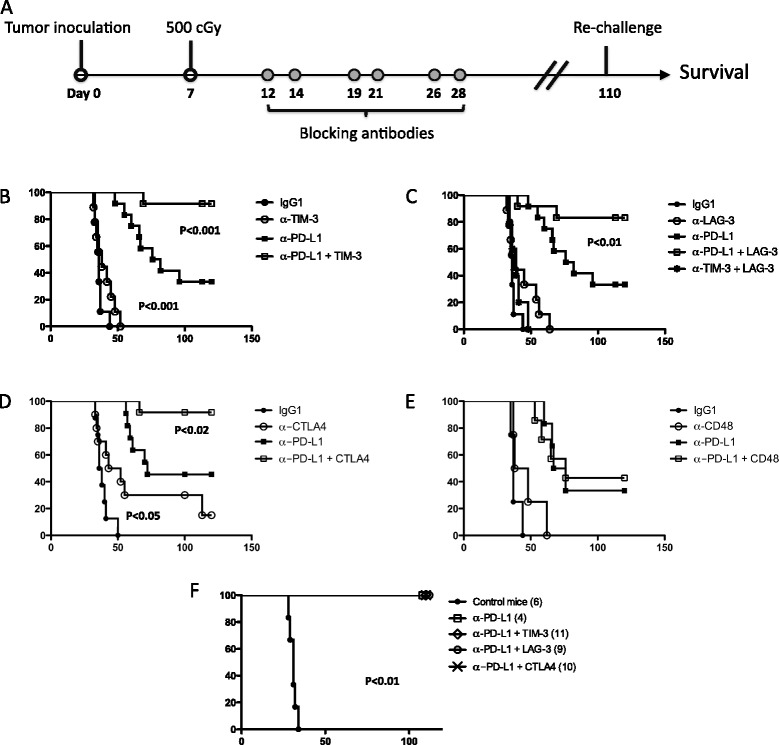
**Blocking of PD-L1 in combination with TIM-3, LAG-3 or CTLA4 after lymphodepleting whole body irradiation synergistically improved survival. (A)** Experimental design: KaLwRij mice received 500 cGy irradiation 7 days after tumor cell injection. Treatment with blocking antibody or control IgG (200 μg ip) was initiated 5 days later and specifically given 12, 14, 19, 21, 26, and 28 days after tumor injection. Survival curves of mice treated with **(B)** anti-TIM-3 only, or in combination with anti-PD-L1, **(C)** anti-LAG-3 only, or in combination with anti-PD-L1, **(D)** anti-CTLA4 only, or in combination with anti-PD-L1, **(E)** anti-CD48 only, or in combination with anti-PD-L1. Survival was compared with control antibody treated mice or mice treated with anti-PD-L1 only. Survival curves represent combined data from three **(B, C, D)** or two **(E)** independent experiments; n = 10-15 mice per experimental group. **(F)** Some of the survivors from panels **B**, **C** and **D** were re-challenged with 1×10^6^ 5T33 myeloma cells on day 110. P values were determined by the log-rank test.

Since multiple immune checkpoint proteins are upregulated on T cells in myeloma-bearing mice (Figure 1), and blocking the PD-L1/PD-1 axis in lymphodepleted animals induces increased expression of LAG-3, TIM-3 and 2B4 checkpoint proteins (Figure 2), we hypothesized that anti-myeloma immunity would be enhanced by blocking combinations of immune checkpoints. Mice were inoculated with 2×10^6^ 5T33 tumor cells iv, received 500 cGy WBI 7 days after tumor cell inoculation, and were treated with checkpoint blocking antibodies (200 ug of each antibody ip) at the time points indicated in Figure 3A. Administration of anti-PD-L1 alone eliminated myeloma in ~40% of mice (Figure 2B-E), which is consistent with our previous results [39]. While treatment with anti-TIM-3 or anti-LAG-3 alone had no affect on survival (Figure 2B, C), co-administration of either antibody with anti-PD-L1 synergistically improved survival rates to greater than 80% (Figure 2B, C). The combination of anti-TIM-3 with anti-LAG-3 failed to improve survival (Figure 2C). Therefore, PD-L1 blockade was necessary in order to see any survival benefit from blocking the two other checkpoints. Survival was also significantly improved by combining anti-PD-L1 with anti-CTLA4 (Figure 2D). Anti-CTLA4 alone also facilitated the elimination of myeloma in approximately 15% of animals. Since a blocking antibody to 2B4 was not available, an antibody to the 2B4 ligand, CD48, was used to block the 2B4/CD48 axis. As shown in Figure 3E, anti-CD48 administered alone or with anti-PD-L1 failed to have any impact on the elimination of myeloma.

In order to test if survivors from the experiments in Figure 3B-D had developed anti-tumor memory, they were challenged with 1×10^6^ 5T33 tumor cells iv 100-110 days after the initial tumor cell inoculation and followed for tumor development. All survivors of the experiments in Figure 3B-D survived the 5T33 re-challenge, indicating that blocking the indicated combinations of checkpoint receptors does not compromise anti-tumor immune memory (Figure 3F).

### Combined checkpoint blockade increases the frequency of myeloma-reactive CD8 and CD4 T cells

**Figure 4:**
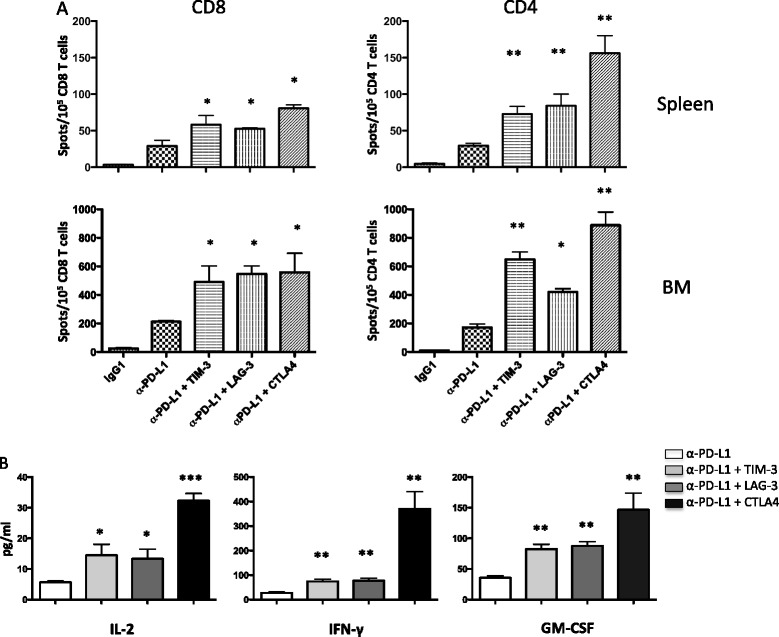
**The frequencies of tumor-reactive spleen and bone marrow-derived CD8 and CD4 T cells were increased in mice treated with a combination of checkpoint blockade.** The experimental design shown in Figure 3 was used. Myeloma bearing mice were treated with 3 doses of control IgG, anti-PD-L1 only, or the combination of anti-PD-L1 with anti-TIM-3, or anti-LAG-3 or anti-CTLA4. **(A)** T cells were isolated from spleens (top row) and bone marrow (bottom row) 21 days after tumor cell injection (i.e., 14 days after irradiation). CD4^+^ and CD8^+^ T cells were purified by immunomagnetic cell sorting and were tested for IFN-γ production in ELISPOT assays using 5T33-CIITA MHC class II^+^ or MHC class II^−^ 5T33 wild-type cells as stimulators, respectively. Purified CD8^+^ or CD4^+^ T cells for each group were pooled from 5-7 individual mice. The graphs are representative of three independent experiments. **(B)** Purified splenic CD8 T cells were stimulated with 5T33 tumor cells for 48 hours. Supernatants were collected and cytokine levels were determined using a multiplex cytokine assay. The graphs are representative of two independent experiments in which the CD8^+^ T cells for each group were pooled from 5 individual mice. For A and B: *p < 0.05, **p < 0.01 as compared with T cells from mice treated with anti-PD-L1 alone.

Since previous data from our laboratory demonstrated that the increased survival of myeloma-bearing mice treated with lymphodepleting WBI and anti-PD-L1 is T cell mediated [39], we set out to determine if combined checkpoint blockade increases numbers of functional tumor-reactive T cells. To do this, mice were treated according to the schedule in Figure 3A, but instead of 6 doses of blocking antibody, they received 3 doses on days 12, 14 and 19 following tumor cell injection. Twenty-one days after tumor cell injection, T cells were harvested from the spleens and bone marrow and CD4 and CD8 T cells were enriched by immunomagnetic cell sorting. Frequencies of tumor-reactive IFN-γ-producing cells were then assessed in ELISPOT assays. CD8 T cells were stimulated with wild-type 5T33 cells, while MHC class II-expressing 5T33 cells (engineered to express CIITA) were used to stimulate CD4 T cells. Both spleen and bone marrow-derived CD4 and CD8 T cells showed a significant increase in tumor-specific IFN-γ producing cells when mice were treated with anti-PD-L1 in combination with anti-TIM-3, or anti-LAG-3 or anti-CTLA4 as compared to anti-PD-L1 alone (Figure 4A).

To determine if there were differences in bulk cytokine production, splenic CD8 T cells were co-cultured with 5T33 tumor cells for 48 hours, supernatants were collected, and cytokine secretion was determined using a multiplex cytokine assay. Similar to results of the IFN-γ ELISPOT assays, there was a significant increase in type 1 cytokines (IL-2, IFN-γ and GM-CSF) produced by CD8 T cells harvested from mice that had received anti-PD-L1 in combination with anti-TIM-3, or anti-LAG-3 or anti-CTLA4 as compared to anti-PD-L1 alone (Figure 4B). Notably, CD8 T cells from mice treated with anti-PD-L1 in combination with anti-CTLA4 produced at least 2-fold higher concentrations of cytokines as compared to mice treated with anti-PD-L1 in combination with anti-TIM-3 or anti-LAG-3.

### Combined checkpoint blockade results in increased expression of PD-1 on T cells, and ongoing PD-L1 blockade in vitro results in elevated numbers of IFN-γ producing cells

**Figure 5:**
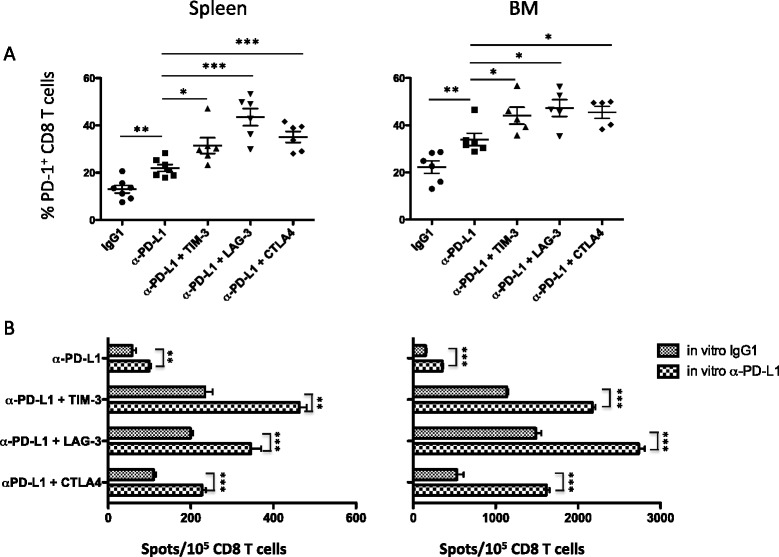
**Combined blockade of immune checkpoint proteins increased PD-1 expression on CD8 T cells and increased frequency of tumor specific CTL.** The experimental design in Figure 3 was used. Mice were treated with 3 doses of control IgG, anti-PD-L1 only or the combination of anti-PD-L1 with anti-TIM-3, or anti-LAG-3 or anti-CTLA4. **(A)** CD8^+^ T cells were isolated from spleens and bone marrow 21 days after tumor cell injection and analyzed for PD-1 expression by flow cytometry. **(B)** IFN-γ produced by CD8 T cells was determined by IFN-γ ELISPOT assays. T cells were incubated with tumor cells and 10 μg/ml IgG1 or tumor cells and 10 μg/ml anti-PD-L1. The graphs represent 2 independent experiments in which the CD8 T cells for each group were pooled from 5 to 7 individual mice. P values were determined by the Student’s t test. *p < 0.05, **p < 0.01, ***p < 0.001.

There is data suggesting that expression of inhibitory receptors, including PD-1, correlates with T cell activation and/or differentiation rather than exhaustion [40]. Other investigators have also shown that the majority of vaccine-induced CD8^+^ T cells upregulate PD-1 [41], and PD-1 has been found on clonally expanded tumor-reactive CD8^+^ T cells isolated from tumors [42]. Similarly, we showed that in mice with 5T33 myeloma, PD-1 expression was upregulated only on host T cells capable of recognizing tumor antigens, and not on non-tumor-specific ovalbumin-reactive OT-1 T cells [39]. In sum, these data indicate that PD-1 is a marker of activated tumor-specific T cells in the cancer setting. Based on these observations, we hypothesized that combined immune checkpoint blockade after lymphodepleting WBI would result in increased percentages of T cells that express PD-1, representing increased numbers of myeloma-reactive T cells. Mice were treated as shown in Figure 3A, except they received only the first 3 doses of antibody. CD8 T cells were harvested from the spleen and bone marrow 21 days after tumor injection and were analyzed for PD-1 expression. In support of our hypothesis, we observed significant increases in percentages of PD-1^+^ CD8 T cells in the spleens and bone marrow of mice treated with anti-PD-L1 in combination with anti-TIM-3, anti-LAG-3 or anti-CTLA4 as compared to anti-PD-L1 alone (Figure 5A).

To explain the increased anti-myeloma immunity after WBI and checkpoint blockade, we have proposed the following model: (a) PD-1^+^ tumor-reactive CD8 T cells are rendered dysfunctional upon encounter with PD-L1 and other checkpoint ligands on the myeloma cells (5T33 expresses high levels of PD-L1) [39] or other cells in the microenvironment, (b) that the PD-1^+^ cells are able to recover function after WBI due to the transient lymphopenic state through mechanisms yet to be identified, and (c) ongoing checkpoint blockade is required to maintain function of the re-activated T cells. To address the last part of this model, we again performed IFN-γ ELISPOT assays on CD8 T cells isolated from mice treated with 500 cGy WBI and combined checkpoint blockade, but anti–PD-L1 or control IgG1 antibody was also added in vitro to the 48-hour CD8 T cell/tumor cell co-cultures. PD-L1 blockade in vitro significantly increased the frequencies of IFN-γ-producing CD8 T cells from mice treated with anti-PD-L1 only or combinations of anti-PD-L1 and other checkpoint blocking antibodies (Figure 5B). These results highlight the importance of the PD-1/PD-L1 pathway in suppressing immunity to the 5T33 myeloma, and they support our model that ongoing checkpoint blockade is needed to maintain the function of activated tumor-specific CD8 cells long enough for them to eliminate the myeloma cells and generate memory.

### Spontaneous and tumor-specific production of Th1 and Th2 cytokines is elevated from CD4 T cells of mice treated with anti-PD-L1 plus anti-CTLA4

**Figure 6:**
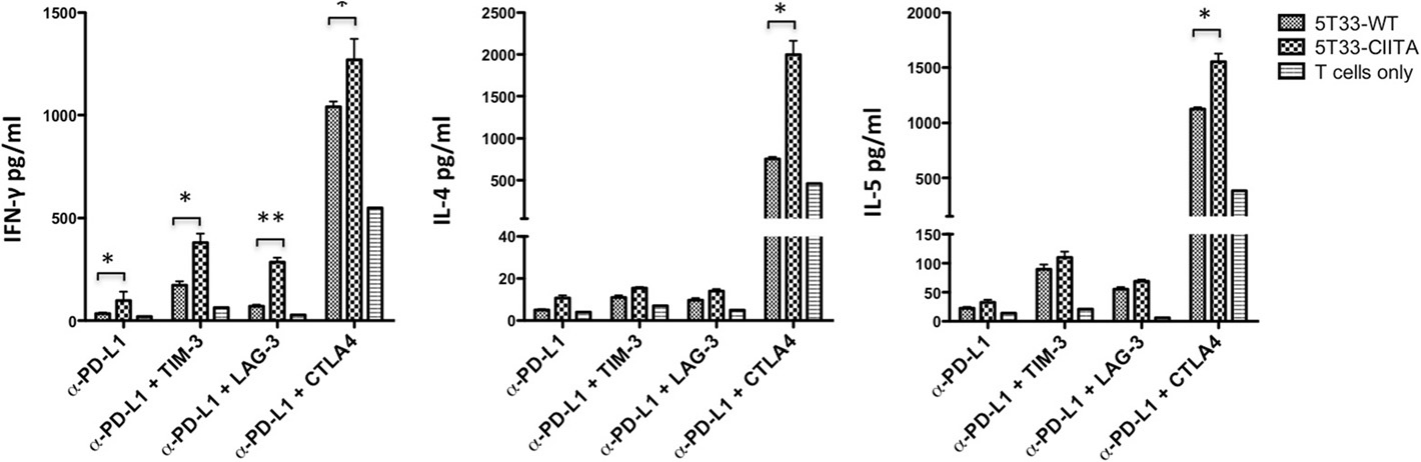
**There is spontaneous release of Th1 and Th2 cytokines from splenic CD4 T cells harvested from mice treated with anti-PD-L1 and anti-CTLA4.** The experimental design shown in Figure 3 was used. Mice were treated with anti-PD-L1, or the combination of anti-PD-L1 with anti-TIM-3, or anti-LAG-3 or anti-CTLA4. T cells were isolated from spleens 21 days after tumor cell injection (14 days after irradiation). CD4 T cells were purified by immunomagnetic cell sorting, then stimulated with MHC class II^−^ 5T33 wild-type cells, 5T33-CIITA MHC class II^+^ cells or T cells only for 48 hours. Supernatants were collected and cytokine levels from were determined using a multiplex cytokine assay. The graphs are representative of two independent experiments in which the CD4^+^ T cells for each group were pooled from 5 individual mice. p < 0.05, **p < 0.01.

CD4 T cells from mice treated with WBI and immune checkpoint blockade were also analyzed for cytokine production in response to tumor cells in vitro. Mice were once again treated according to the schedule in Figure 3A, except they received three antibody doses instead of six. On day 21 after myeloma inoculation, CD4 T cells were harvested from the spleen and purified by immunomagnetic cell sorting. Cells were incubated with 5T33-CIITA tumor cells expressing MHC class II molecules for 48 hours followed by cytokine analysis in multiplex cytokine assays. Spontaneous cytokine release was analyzed by incubating CD4 T cells alone or with wild-type 5T33 cells. CD4 T cells harvested from mice treated with anti-PD-L1 plus anti-CTLA4 spontaneously released IFN-γ, as well as the Th2 cytokines IL-4 and IL-5 (Figure 6). Cytokine release was significantly increased when the T cells were incubated with 5T33-CIITA MHC class II^+^ tumor. CD4 T cells harvested from mice treated with anti-PD-L1 plus anti-LAG-3 or anti-TIM-3 also released IFN-γ when stimulated with 5T33-CIITA cells, but there was no spontaneous or tumor-induced release of IFN-γ, IL-4 and IL-5.

## Discussion

From our results to date, we propose a working model whereby low dose WBI generates a transient state of lymphopenia, which allows for the reactivation of myeloma-reactive T cells that have been rendered dysfunctional by checkpoint proteins. Checkpoint blockade then allows the re-activated T cells to remain functional and eliminate the myeloma. Lymphodepleting WBI and PD-L1 blockade failed in two solid tumors, but provided anti-tumor efficacy in other murine hematologic malignancy models, suggesting that hematologic malignancies may be more amenable to this treatment strategy [39]. The current study furthers the earlier work by demonstrating that myeloma experienced T cells upregulate expression of multiple checkpoint receptors including PD-1, LAG-3, TIM-3 and 2B4. Furthermore, treatment of myeloma-bearing mice with lymphodepletive WBI and dual checkpoint blockade induced a synergistic anti-myeloma effect, and this enhanced elimination of myeloma was associated with increased numbers of IFN-γ-producing tumor-reactive T cells and elevated cytokine production by T cells in response to tumor antigens.

Multiple immune checkpoints have been previously shown to be upregulated on T cells in several murine solid tumor models, and targeting more than one pathway has demonstrated increased anti-tumor efficacy [19,21,22,43-52]. However, there is a paucity of literature regarding expression of immune checkpoints on T cells in hematologic malignancies or the effect of targeting more than one checkpoint as therapy for these cancers [22]. Clinically, PD-1 and CTLA4 have been simultaneously targeted for the treatment of melanoma, and anti-tumor activity appears to be more robust than targeting each pathway alone [15]. Notably, autoimmune manifestations with this combination did not appear to be significantly worsened. In our studies, blockade of the PD-1/PD-L1 pathway appears to provide an activation threshold for myeloma-reactive T cells that allows blockade of other checkpoints to provide synergistic anti-myeloma responses. Interestingly, blockade of LAG-3, TIM-3 or CTLA4 alone had only modest or no effect on elimination of myeloma after WBI. How inhibitory signals transmitted through TIM-3 and LAG-3 synergize with those transmitted through PD-1 is unknown. It could be that there is constitutive inhibitory signaling through TIM-3 and LAG-3 by the presence of pleotropic ligands (Additional file 2: Figure S2). The blocking of these constitutive inhibitory signals may have an observable anti-tumor effect only when dominant inhibitory signaling through PD-1 is blocked. In contrast, when combined with PD-L1 blockade, potent anti-myeloma effects were observed when each of these pathways was targeted (Figure 3).

Treatment with WBI and anti-PD-L1 resulted in increased expression of PD-1 as well as LAG-3, TIM-3 and 2B4 (Figure 2). Furthermore, blockade of PD-L1 with other immune checkpoints (TIM-3, LAG-3 and CTLA4) drove up PD-1 expression on T cells even further (Figure 5A). This result is surprising, as the expression of checkpoint receptors on T cells have been regarded as markers of dysfunction. In settings of chronic antigen exposure, such as cancer, expression of checkpoint receptors such as PD-1, CTLA4, TIM-3, LAG-3, and 2B4 have been associated with dysfunctional T cells, often termed exhausted or tolerized [53-57]. However, there are recent reports that PD-1^+^ T cells may not be functionally impaired, but instead represent T cells that have been activated. In healthy adults, PD-1^+^ peripheral CD8 T cells were shown to be effector memory cells and not exhausted T cells [58]. In support of this data, Baitsch et al. found that the majority of human effector memory T cells co-expressed PD-1, CTLA4, KLRG-1, 2B4, LAG-3 or CD160 [59]. Also, in human CD8 T cells, an increase in co-expression of inhibitory receptors such as PD-1, TIM-3, 2B4, CD160 and KLRG1 correlated with T cell differentiation or activation status [40]. In support of these findings we found that sorted PD-1^+^ tumor-experienced CD8 T cells secrete IFN-γ when in vitro incubated with myeloma cells and anti-PD-L1 blocking antibody (unpublished data). The mechanism(s) by which checkpoint receptors are regulated by blocking antibodies and how this induces T cell activation is currently unknown. In order to dissect the mechanism(s) involved, further investigation of the myeloma-reactive T cells is required. Since the tumor antigens in the 5T33 tumor model are unknown, we have genetically modified 5T33 cells to express a model antigen, SIINFEKL ovalbumin peptide, so that we can detect and isolate ‘tumor-specific’ cells using a MHC/SIINFEKL pentamer reagent. Once myeloma-specific CD8 T cells are identified and isolated, the mechanism of how checkpoint blockade regulates checkpoint expression can be interrogated.

Currently, there is no reliable way to predict which patients are going to benefit from checkpoint blockade. However, our data may provide clues as to which combination of checkpoint molecules to block based on the expression pattern of checkpoint receptors on T cells. We observed increased expression of TIM-3, LAG-3, CTLA4, and 2B4 on both CD8 and CD4 T cells in myeloma-bearing mice, and anti-myeloma synergy occurred when the PD-1/PD-L1 axis was blocked in combination with blocking TIM-3, LAG-3, or CTLA4. Despite upregulation of 2B4 on T cells with increasing myeloma burden, which has also been observed on CD8 and CD4 T cells in a mouse pancreatic cancer model [60], blockade of PD-L1 plus the ligand for 2B4, CD48, did not provide any additional benefits over PD-L1 blockade alone. It is possible that the CD48-specific antibody used in this study did not block the 2B4/CD48 receptor axis, although the antibody clone used has been reported to block the pathway [61]. Another possible reason for the lack of synergy stems from a report that 2B4 has both T cell proliferative and inhibitory effects [32]. Recently, CD48 was found to be expressed on more than 90% of plasma cells from myeloma patients at higher levels than those observed on normal lymphocytes [62], although it is unknown whether this elevated expression could have a negative impact on T cell reactivity. This will need to be examined more closely in future studies.

As noted earlier, we observed anti-myeloma synergy when mice were treated with anti-PD-L1 and anti-CTLA4 blocking antibodies. In clinical trials, treatment with anti-CTLA4 has been associated with multiple immune-related adverse events including colitis, hepatitis, and thyroiditis [63]. Treatment of patients with antibodies targeting the PD-1/PD-L1 pathway has also resulted in some toxicities, although they have typically been less severe than those observed with CTLA4 antibodies [64]. We did not observe weight loss or any gross toxicities in mice treated with anti-PD-L1 and anti-CTLA4, despite the fact that this checkpoint blockade combination produced significantly more Th1 and Th2 cytokines than anti-PD-L1 in combination with anti-TIM-3 or anti-LAG-3 (Figures 4B and 6). Notably, the frequencies of IFN-γ-producing tumor-reactive CD8 T cells from anti-PD-L1/CTLA4 treated mice were similar to those treated with the anti-PD-L1/LAG-3 and anti-PD-L1/TIM-3 combinations (Figures 4A and 5B). However, since the bulk production of IFN-γ was increased (Figure 4B), it appears that tumor-reactive CD8 T cells in anti-PD-L1/CTLA4 treated mice produce more cytokines on a per cell basis. Similar to this observation, combined immunotherapy using OX40 stimulation with CTLA4 inhibition enhanced Th1 and Th2 cytokine production by effector T cells [65]. T regulatory function was not inhibited by this approach. In our study, there were no differences in the percentages of Gr-1^+^CD11b^+^ myeloid cells or regulatory T cells in the spleens of myeloma-bearing mice treated with anti-PD-L1 plus anti-CTLA4 as compared to anti-PD-L1 plus other checkpoint antibodies (Additional file 3: Figure S3). These data suggest that these cells may not be involved with regulating T cell cytokine production. However, confirmatory functional studies remain to be done. It is possible that anti-tumor synergy produced by anti-PD-L1 in combination with anti-LAG-3 or anti-TIM-3 antibodies may be a less toxic alternative to anti-PD-L1 in combination with anti-CTLA4 antibodies.

Finally, it is important to note that we previously reported treatment with a lymphodepleting dose of 500 cGy WBI prior to treatment with anti-PD-L1 was a prerequisite for generating effective anti-myeloma immunity [39]. Therefore, in the current study we also treated mice with 500 cGy WBI prior to administering checkpoint-blocking antibodies. The mechanisms of how WBI sensitizes the immune system to produce effective checkpoint blockade has yet to be determined. Multiple immune factors may be important such as providing a lymphopenic environment to induce homeostatic expansion of tumor-reactive T cells.

## Conclusions

In summary, we show that combined immune checkpoint blockade provides a synergistic anti-myeloma effect in mice treated with low dose, lymphodepleting WBI. The anti-myeloma effect correlates with activation of T cells, which appears to be maintained by checkpoint blockade. The importance of sustaining immune checkpoint blockade until tumor cells are eliminated is highlighted by the positive impact addition of anti-PD-L1 had on IFN-γ-producing T cells when added to T cell/myeloma cell co-cultures in ELISPOT assays (Figure 5B). Increased T cell expression of checkpoint molecules following checkpoint blockade suggests that expression of these molecules is an indicator of T cell activation, rather than a state of irreversible exhaustion. Given our anti-myeloma results using checkpoint blockade, continued study of checkpoint blockade in other hematologic malignancies is warranted. In future studies, it will be interesting to see if targeting more than two checkpoint pathways simultaneously in myeloma and other hematologic malignancies can further improve anti-tumor immunity without generating unacceptable autoimmunity. Understanding the mechanisms of tumor cell elimination induced by combined checkpoint blockade and low dose WBI, and determining if other lymphodepleting strategies can be used, such as lymphodepleting drugs or low doses of T cell depleting antibodies, will help to expand the translational applications of this approach. Finally, this therapeutic approach could serve as a platform for other immune therapies, including T cell adoptive transfer.

## Methods

### Mice

C57BL/KaLwRij (KaLwRij) and (KaLwRij × C57BL/6.SJL)F1 mice were bred and housed in the Medical College of Wisconsin Biomedical Resource Center, which is an American Association for the Accreditation of Laboratory Animal Care (AAALAC)–accredited facility. All animal work was reviewed and approved by the Medical College of Wisconsin Institutional Animal Care and Use Committee.

### Tumor cells

The 5T33 murine myeloma cell line was derived from a myeloma that spontaneously arose in a C57BL/KaLwRij mouse [40]. 5T33 cells were transduced to express emerald green fluorescent protein (5T33-GFP), as previously described [39]. MHC class II^+^ 5T33 cells (designated 5T33-CIITA) were derived by transducing 5T33 cells with a lentiviral expression vector (PLVX-N1; ClonTech, Mountain View, CA) encoding the MHC class II transactivator (CIITA) gene. Mice were inoculated with 2×10^6^ 5T33 or 5T33-GFP cells intravenously (iv). Myeloma-bearing mice were considered as moribund and euthanized when they developed paraparesis or paraplegia. Occasionally, 5T33-injected mice developed tumor masses or other related lesions and were euthanized when the size of the mass or lesion exceeded 250 mm^2^. Other symptoms of advanced tumor burden included splenomegaly, hepatomegaly, or neurologic impairment.

### Antibodies and flow cytometry

The following monoclonal anti-mouse antibodies and flow cytometry reagents were obtained from eBioscience (San Diego, CA): anti-CD4 (GK1.5), anti-CD8 (53-6.7), anti-PD-1 (J43), anti-TIM-1 (RMT1-4), anti-TIM-3 (RMT3-23), anti-LAG-3 (C987W), anti-2B4 (244F4), anti-CTLA4 (UC10-4F10-11), anti-BTLA (8F4), anti-PD-L1 (MIH5), anti-galectin-9 (108A2), anti-I-A^b^ (AF6-120.1), anti-CD80 (16-10A1), anti-H2Kb (AF6-88.5.5.3), anti-CD11b (M170), anti-CD11c (N418), anti-F4/80 (BM8), anti-Gr-1 (RB6-8C5), anti-Foxp3 (FJK-16 s) and propidium iodide staining solution. The following antibodies and reagents were obtained from Biolegend (San Diego, CA): anti-CD8 (53-6.7), anti-PD-1 (J43), and anti-TIM-3 (B8.2C12), anti-CD19(GD5). Flow cytometry was done on a BD Biosciences (Franklin Lakes, NJ) LSRII flow cytometer, and resulting data analyzed using FlowJo software (Tree Star, Inc.).

### Antibody treatment of myeloma-bearing mice

C57BL/KaLwRij or (KaLwRij × C57BL/6.SJL)F1 mice were injected with 2×10^6^ 5T33 cells iv. The myeloma bearing mice were irradiated with 500 cGy whole body irradiation (WBI) using a cesium irradiator 7 days after myeloma inoculation. Antibody treatment was initiated 5 days after WBI and administered on days 12, 14, 19, 21, 26, and 28 after myeloma inoculation. 5T33 tumor-bearing mice were treated with anti-PD-L1 (clone 10F.9G2; BioXCell), anti-LAG-3 (clone C9B7W), anti-TIM-3 (clone 5D12; CoStim/Novartis), anti-CTLA4 (clone 9H10; BioXCell), or anti-CD48 (clone HM48-1; BioXCell) monoclonal antibodies at the indicated time points. Some mice received a combination of anti-PD-L1 plus anti-LAG-3, anti-TIM-3 or anti-CTLA4. Rat immunoglobulin G (IgG) was administered as control antibody. All antibodies were given at a dose of 200 μg by intraperitoneal (ip) injection. Myeloma-bearing mice were considered as moribund and euthanized when they developed hind-leg paralysis or other defined endpoints. Mice that survived the initial treatment were re-challenged with 1×10^6^ 5T33 tumor cells 100-110 days after the first inoculation.

### Interferon-gamma (IFN-γ) ELISPOT assays

To assess for presence of tumor-reactive, interferon-gamma (IFN-γ)-secreting CD8 or CD4 T cells, T cells were harvested from the spleen and bone marrow, and isolated by immunomagnetic cell sorting as previously described [35]. IFN-gamma enzyme-linked immunosorbent spot (ELISPOT) assays were done using mouse IFN-γ ELISPOT kits from BD Biosciences, as described previously [39]. The ELISPOT data was quantified using a Cellular Technology Limited (CTL) ImmunoSpot Analyzer (CTL Analyzers, Cleveland, OH).

### Bio-plex cytokine assays

CD4 and CD8 T cells from antibody treated myeloma-bearing mice were cultured in media alone or in the presence of 5T33 wild type or 5T33-CIITA tumor cells. Culture supernatants were harvested after 48 hours and stored at −80°C. Thawed supernatants were then analyzed using a murine multiplex cytokine kit (Bio-Rad, Hercules, CA) to detect IL-2, IL-4, IL-5, IL-10, IL-12p70, granulocyte-macrophage colony stimulating factor (GM-CSF), tumor necrosis factor-alpha (TNF-α), and IFN-γ. Cytokines were quantified using a Bio-Plex protein 200 array reader, and data was automatically processed and analyzed by the Bio-Plex Manager Software 4.1 using standard curves generated from recombinant cytokine standards. All samples were assayed in duplicate.

### Statistics

Survival curves were compared using the log-rank (Mantel Cox) test. Other experiments were compared using the Student’s t test. P values <0.05 were considered as significant. Statistical analysis was done using Prism version 5.0a software (GraphPad Software, La Jolla, CA).

## Additional files

Additional file 1: Figure S1. Expression of immune checkpoint proteins on T cells in spleens of myeloma bearing mice over time. KaLwRij mice were inoculated with 2×10^6^ 5T33-GFP cells iv. Myeloma bearing mice were euthanized between days 7 and 28 and when moribund at days 29-40 after myeloma injection. Spleens were harvested and CD4^+^ and CD8^+^ T cells were analyzed for immune checkpoint protein expression over time by flow cytometry. T cells harvested from naïve nonmyeloma bearing mice were analyzed as controls. Immune checkpoint protein percentages were calculated based on isotype controls. *p < 0.05, **p < 0.01, ***p < 0.001 as compared to T cells from naïve non-myeloma bearing mice. Additional file 2: Figure S2. Membrane expression of immune checkpoint protein ligands on myeloma and splenocytes. **(A)** PD-L1, galectin-9, MHC class II (I-A^b^) and CD80 expression on 5T33 tumor cells (solid line), and on 5T33 tumor cells irradiated in vitro with 500 cGy (dashed line). Isotype controls are shaded in gray. **(B)** Expression of PD-L1, galectin-9, MHC class II (I-A^b^) and CD80 on B cells, macrophages, dendritic cells (DC) and monocytes in the spleens of moribund myeloma-bearing mice. Fluorescence minus one (FMO) controls are shaded in gray. Additional file 3: Figure S3. Percentages of myeloid and regulatory T cells in the spleens of mice treated with combinations of blocking antibodies to immune checkpoint proteins. Mice were treated as shown in Figure 3A. Myeloma bearing mice were treated with three doses of control IgG, anti-PD-L1 only, or a combination of anti-PD-L1 with anti-TIM-3, anti-LAG-3 or anti-CTLA4. At day 21 after myeloma inoculation, spleens were harvested and the percentages of Foxp3^+^CD4^+^ regulatory T cells and Gr-1^+^CD11b^+^ myeloid cells were analyzed by flow cytometry.

## Abbreviations

CTLA4: Cytotoxic T-lymphocyte antigen-4
CIITA: MHC class II transactivator
GFP: Green fluorescent protein
GM-CSF: Granulocyte-macrophage colony-stimulating factor
IFN: Interferon
LAG-3: Lymphocyte activating gene-3
PD-1: Programmed death receptor-1
PD-L1: Programmed death receptor ligand-1
TIL: Tumor-infiltrating lymphocyte
TIM-3: T cell immunoglobulin and mucin domain-3
WBI: Whole body irradiation
