# Supplementary figures and images for: Combined immune checkpoint protein blockade and low dose whole body irradiation as immunotherapy for myeloma
Source: J Immunother Cancer. 2015 Jan 20;3:2. doi: 10.1186/s40425-014-0043-z (PMC4302511; doi:10.1186/s40425-014-0043-z)

Figure S1

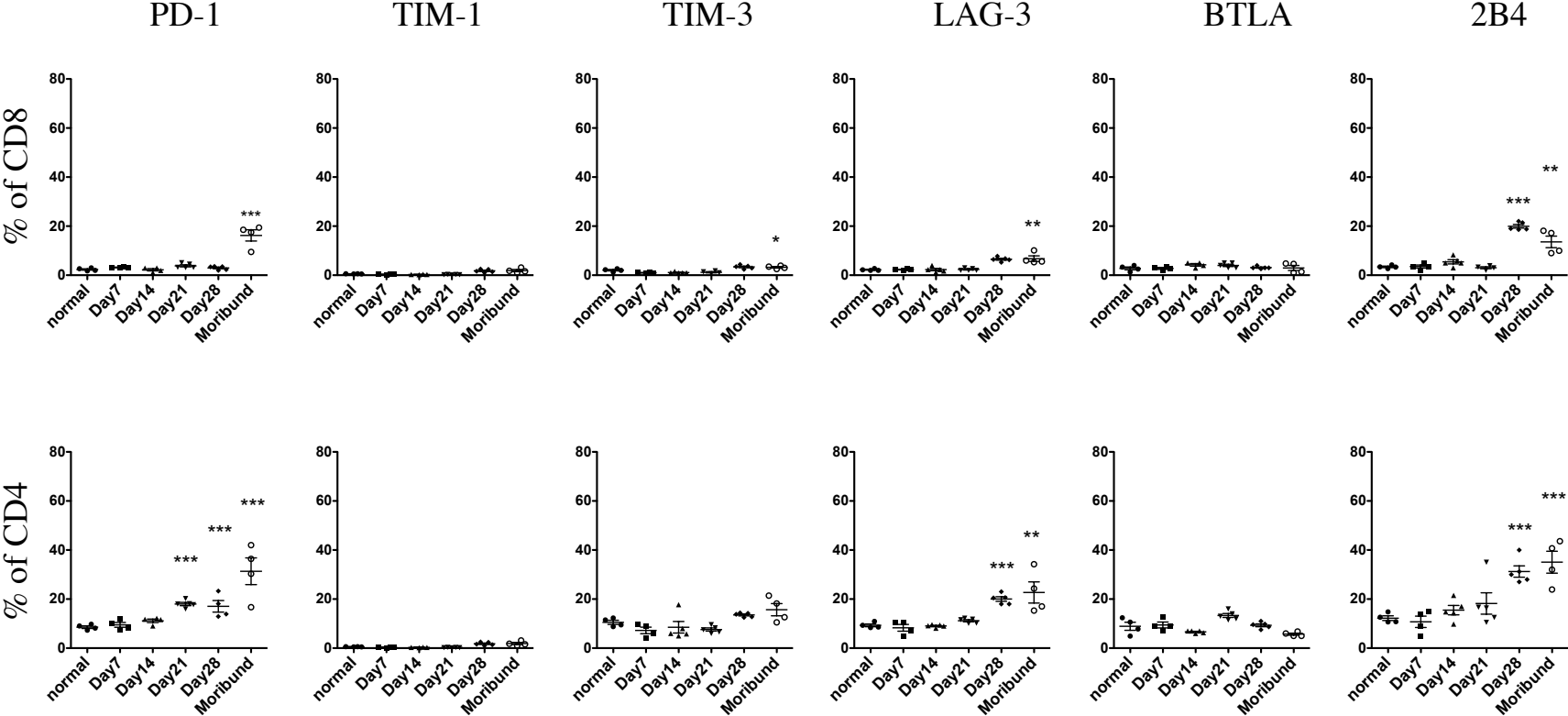

Supplement: Additional file 1: Figure S1. — Expression of immune checkpoint proteins on T cells in spleens of myeloma bearing mice over time. KaLwRij mice were inoculated with 2×10^6^ 5T33-GFP cells iv. Myeloma bearing mice were euthanized between days 7 and 28 and when moribund at days 29-40 after myeloma injection. Spleens were harvested and CD4+ and CD8+ T cells were analyzed for immune checkpoint protein expression over time by flow cytometry. T cells harvested from naïve nonmyeloma bearing mice were analyzed as controls. Immune checkpoint protein percentages were calculated based on isotype controls. *p < 0.05, **p < 0.01, ***p < 0.001 as compared to T cells from naïve non-myeloma bearing mice. [file 40425_2014_43_MOESM1_ESM.pdf]

Figure S2

A

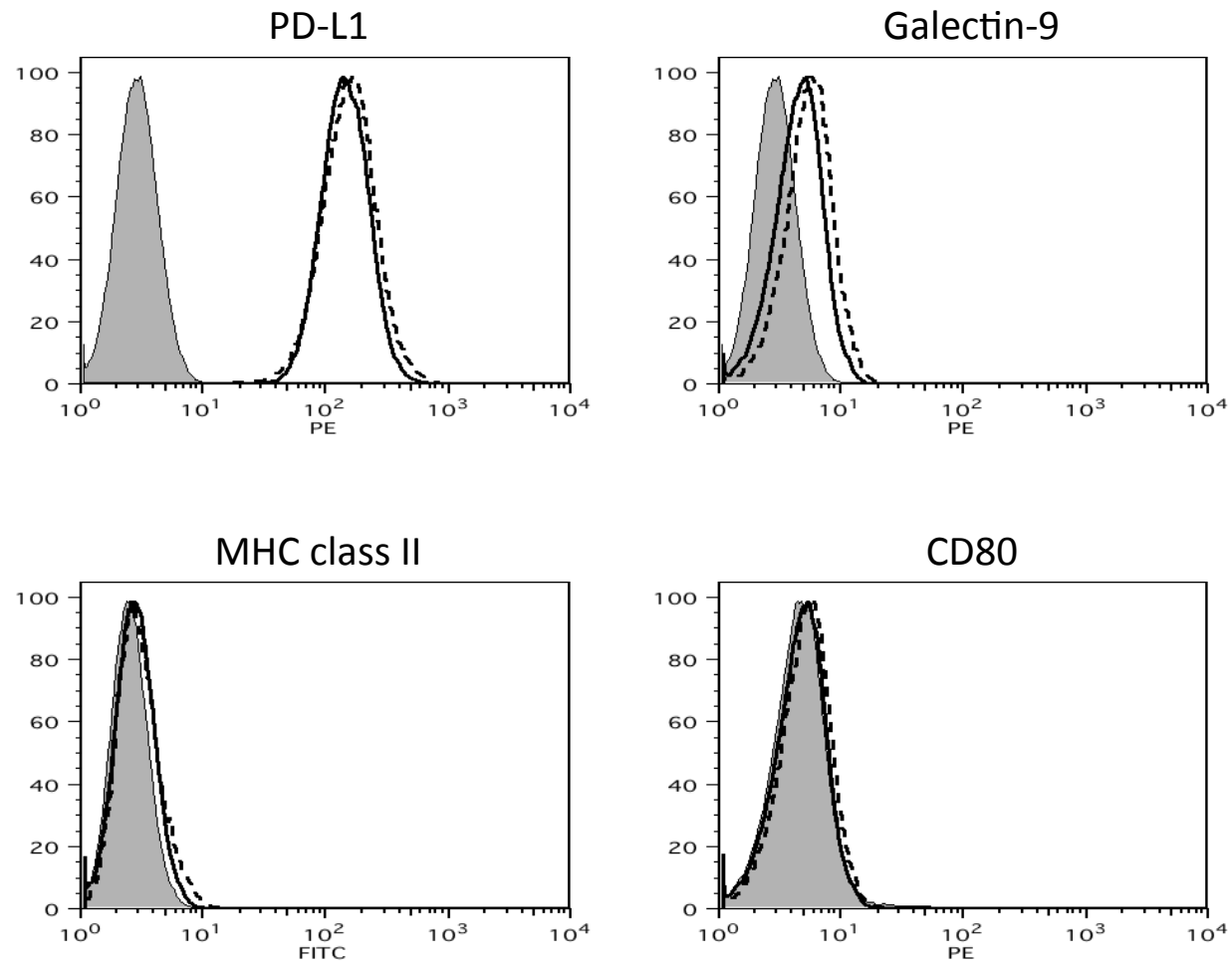

Figure S2

B

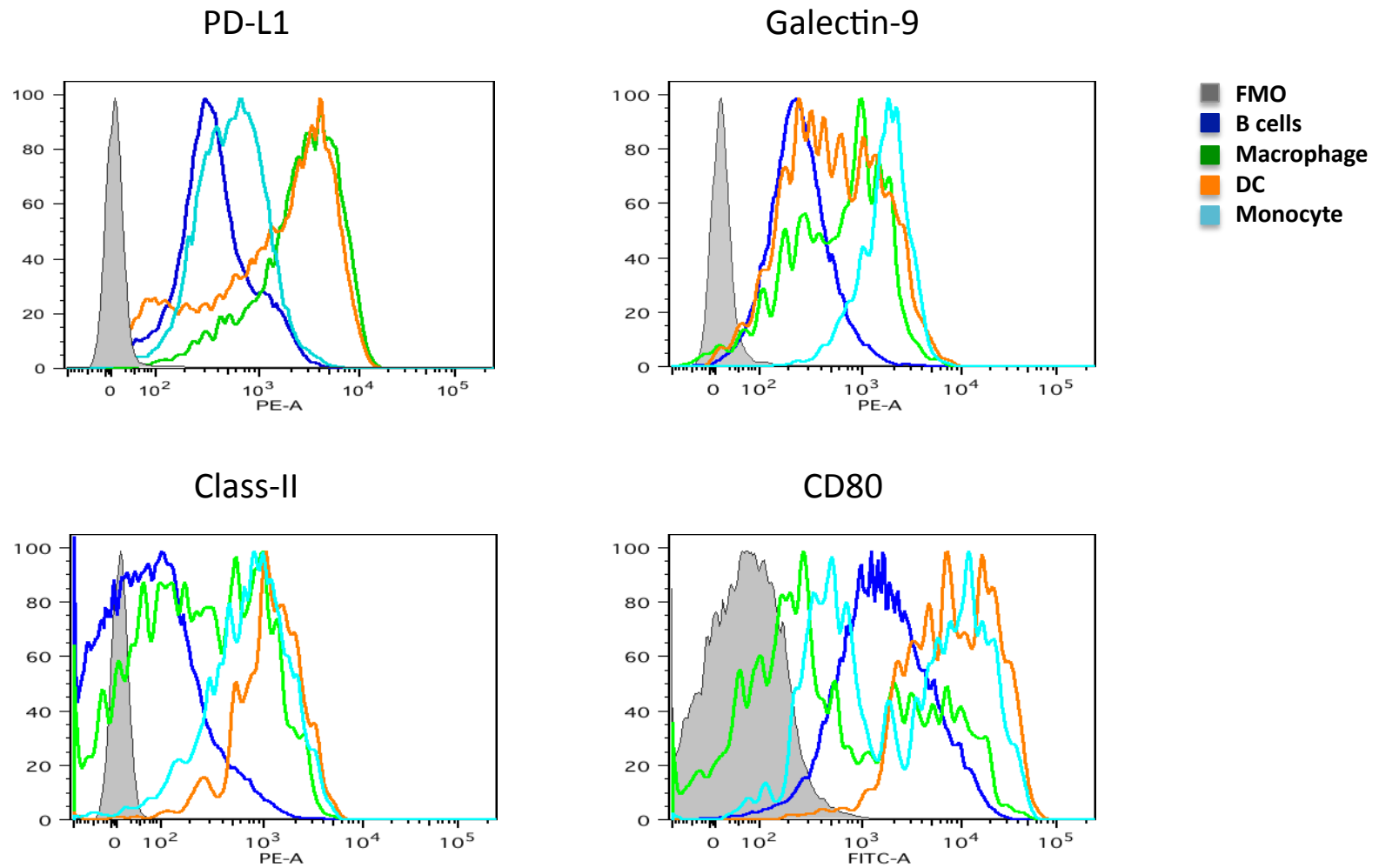

Supplement: Additional file 2: Figure S2. — Membrane expression of immune checkpoint protein ligands on myeloma and splenocytes. (A) PD-L1, galectin-9, MHC class II (I-Ab) and CD80 expression on 5T33 tumor cells (solid line), and on 5T33 tumor cells irradiated in vitro with 500 cGy (dashed line). Isotype controls are shaded in gray. (B) Expression of PD-L1, galectin-9, MHC class II (I-Ab) and CD80 on B cells, macrophages, dendritic cells (DC) and monocytes in the spleens of moribund myeloma-bearing mice. Fluorescence minus one (FMO) controls are shaded in gray. [file 40425_2014_43_MOESM2_ESM.pdf]

Figure S3

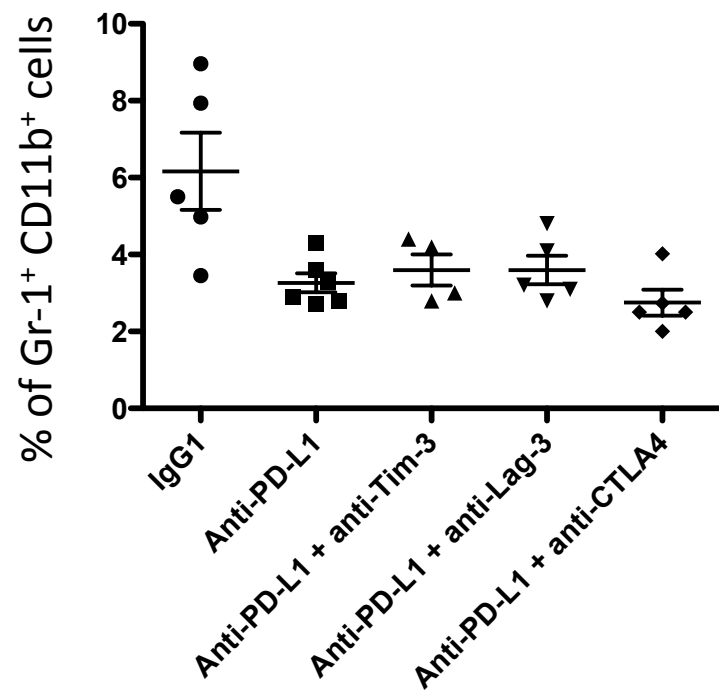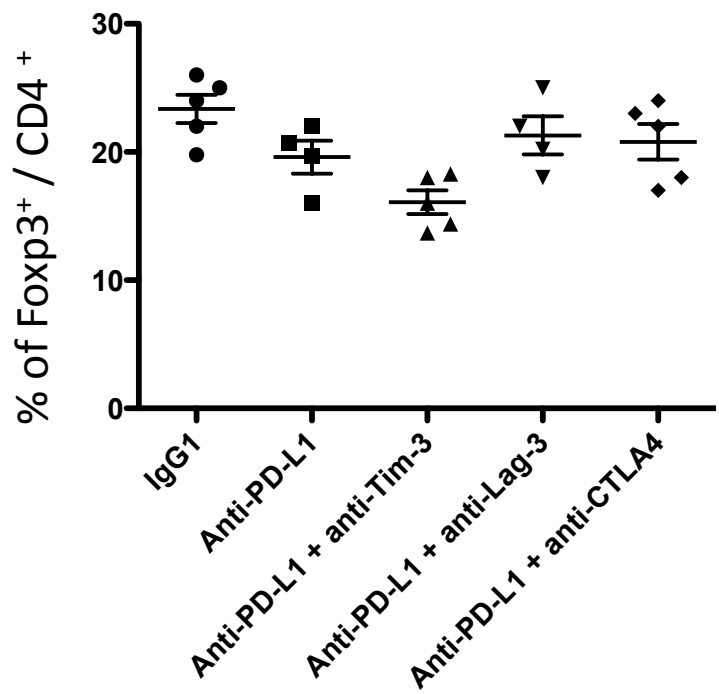

Supplement: Additional file 3: Figure S3. — Percentages of myeloid and regulatory T cells in the spleens of mice treated with combinations of blocking antibodies to immune checkpoint proteins. Mice were treated as shown in Figure 3A. Myeloma bearing mice were treated with three doses of control IgG, anti-PD-L1 only, or a combination of anti-PD-L1 with anti-TIM-3, anti-LAG-3 or anti-CTLA4. At day 21 after myeloma inoculation, spleens were harvested and the percentages of Foxp3+CD4+ regulatory T cells and Gr-1+CD11b+ myeloid cells were analyzed by flow cytometry. [file 40425_2014_43_MOESM3_ESM.pdf]
